# Assessment of genetic diversity and population structure of U.S. Polypay sheep from breed origins to future genomic selection

**DOI:** 10.3389/fgene.2024.1436990

**Published:** 2024-08-05

**Authors:** Carrie S. Wilson, Jessica L. Petersen, Luiz F. Brito, Brad A. Freking, Sara M. Nilson, J. Bret Taylor, Thomas W. Murphy, Ronald M. Lewis

**Affiliations:** ^1^ USDA, ARS, Range Sheep Production Efficiency Research Unit, U.S. Sheep Experiment Station, Dubois, ID, United States; ^2^ Department of Animal Science, University of Nebraska-Lincoln, Lincoln, NE, United States; ^3^ Department of Animal Sciences, Purdue University, West Lafayette, IN, United States; ^4^ USDA, ARS, Livestock Bio-Systems Research Unit, Roman L. Hruska U.S. Meat Animal Research Center, Clay Center, NE, United States

**Keywords:** *Ovis aries*, effective population size, inbreeding, linkage disequilibrium, diversity

## Abstract

Knowledge of past and present genetic diversity within a breed is critical for the design and optimization of breeding programs as well as the development of strategies for the conservation of genetic resources. The Polypay sheep breed was developed at the U.S. Sheep Experiment Station (USSES) in 1968 with the goal of improving productivity in Western U.S. range flocks. It has since flourished in the more intensively managed production systems throughout the U.S. The genetic diversity of the breed has yet to be documented. Therefore, the primary objective of this study was to perform a comprehensive evaluation of the genetic diversity and population structure of U.S. Polypay sheep using both pedigree- and genomic-based methods. Pedigree data from 193 Polypay flocks participating in the National Sheep Improvement Program (NSIP) were combined with pedigree records from USSES (n = 162,997), tracing back to the breed’s origin. A subset of these pedigreed sheep from 32 flocks born from 2011 to 2023 were genotyped with the GGP Ovine 50K BeadChip containing 51,867 single nucleotide polymorphisms (SNPs). Four subgroups were used for the pedigree-based analyses: 1) the current generation of animals born in 2020–2022 (n = 20,701), 2) the current generation with a minimum of four generations of known ancestors (n = 12,685), 3) only genotyped animals (n = 1,856), and 4) the sires of the current generation (n = 509). Pedigree-based inbreeding for the full population was 2.2%, with a rate of inbreeding of 0.22% per generation. Pedigree-based inbreeding, Wright’s inbreeding, and genomic inbreeding based on runs of homozygosity were 2.9%, 1.3%, and 5.1%, respectively, for the genotyped population. The effective population size ranged from 41 to 249 for the pedigree-based methods and 118 for the genomic-based estimate. Expected and observed heterozygosity levels were 0.409 and 0.403, respectively. Population substructure was evident based on the fixation index (F_ST_), principal component analysis, and model-based population structure. These analyses provided evidence of differentiation from the foundation flock (USSES). Overall, the Polypay breed exhibited substantial genetic diversity and the presence of a population substructure that provides a basis for the implementation of genomic selection in the breed.

## Introduction

**FIGURE 1:**
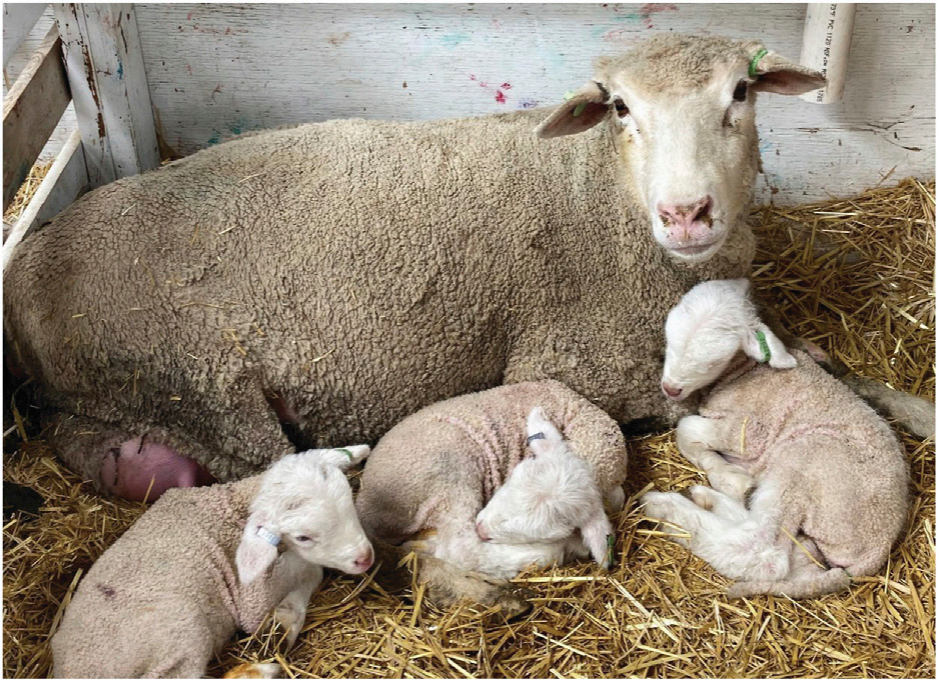
U.S. Sheep Experiment Station Polypay ewe and her triplets.

The Polypay breed was developed in 1968 at the U.S. Sheep Experiment Station (USSES, Dubois, ID, United States). It was developed with equal contributions from Dorset, Rambouillet, Targhee, and Finnsheep. It has since been maintained as a stabilized composite breed that is predicted to retain 75% of maximum individual and maternal heterosis. The five primary selection objectives of the breed development were high lifetime prolificacy, first lambing at 1 year of age, more than one lambing event per year, rapid lamb growth, and high carcass quality (Hulet et al., 1984). An image of a USSES Polypay ewe and her triplets is provided in Figure 1. The foundation breeds were selected to maintain the ruggedness of the Western U.S. range ewe while significantly improving reproductive characteristics. The Targhee and Rambouillet breeds were selected for their hardiness, large body size, flocking instinct, and fleece characteristics. The Polled Dorset was selected for carcass quality, milking ability, and long breeding season. The Finnsheep was selected for early puberty, early *postpartum* fertility, and a high lambing rate (Hulet et al., 1984).

Matings were initiated in 1968 by crossing four unrelated Dorset rams, selected for large frame size and proven lamb productivity, with USSES Targhee ewes and one Finnsheep ram with USSES Rambouillet ewes. The Finnsheep ram was from the original 1968 imports of five rams from Ireland (Oltenacu and Boylan, 1981; Hulet et al., 1984). Four additional Finnsheep rams were added from 1969 to 1972. Beginning in 1969, Dorset-Targhee × Finnsheep-Rambouillet crosses were initiated. By 1970, the 4-breed cross was inter se mated. Development of the foundation lines and two-breed crosses continued for several years (Hulet et al., 1984). Throughout each mating cycle, replacements were retained according to the five primary selection objectives of breed development. Sales of surplus Polypay rams were reported from 1971 to 1974, with the first official Polypay sales in 1975 and the formation of the American Polypay Sheep Association in 1980 (American Polypay Sheep Association, 2024).

The Polypay is polled, with ewe body weight averaging 72 kg (Snowder, 2001). The reported average number of lambs born to mature ewes is 2.4 lambs with a 120-day total litter weight of 63.8 kg. Fiber diameter ranges from 24 to 33 μm, staple length ranges from 7.6 to 12.7 cm, and grease fleece weight ranges from 2.7 to 4.5 kg with a 57% clean fleece yield. An increase in profits over other U.S. breeds of 15%–36% has been reported, primarily driven by improved returns from lamb sales (Snowder, 2001). Further breed refinements have occurred in the more than 20 years of selection that have occurred since this report. Although the breed was initially developed for Western U.S. range production, the Polypay has proven to be versatile in other production and management systems. Throughout the Central and Eastern U.S., the Polypay has been utilized in intensive accelerated lambing systems to produce more than one lamb crop per year (Vanimisetti and Notter, 2012; Hulet and Stellflug, 2019). Although the utilization of Polypay sheep has remained primarily within the U.S., Canada, and Mexico (Stachowicz et al., 2018), it has also been reported as far south as Brazil (McManus et al., 2014). The number of annual lamb records for Polypays included in the National Sheep Improvement Program (NSIP) has averaged 6,900 from 2020 to 2022. In Canada, 267 Polypay sheep were reported in 2022 (Agriculture and Agri-Food Canada, 2024), with smaller numbers in Mexico and Brazil.

Many U.S. sheep breeders participate in genetic evaluation through the NSIP (http://nsip.org), which provides estimated breeding values (EBVs) and selection indexes for a wide range of production traits (Notter, 1998). Novel attributes are currently being evaluated to enable U.S. sheep breeders to improve their flocks for robustness and climatic resilience traits that are of economic relevance and include lamb survival, gastrointestinal parasitism, and ewe longevity (Burton et al., 2023; Lewis, 2023). The Polypay breed represents the “semi-prolific” category of this effort to evaluate novel traits and includes the recruitment of Polypay breeders highly engaged in the use of EBVs and moving toward the implementation of routine genomic evaluations. Hanna et al. (2023) reported that the Polypay had the shortest productive life of the four breeds evaluated (Columbia, Rambouillet, and Targhee), indicating the need for genetic improvement of survival and longevity traits in the breed.

Selection decreases genetic variation in a population. As the seedstock industry moves toward the implementation of genomic selection, the potential for substantial increased genetic gain is accompanied by a loss of genetic diversity (van der Werf et al., 2014; Kristensen et al., 2015). Prior to the implementation of genomic selection, establishing a baseline of the current genetic diversity of the Polypay breed is necessary. Therefore, the primary objectives of this study were to assess the current genetic diversity and the population structure of the Polypay breed in the founder and other U.S. flocks using both pedigree- and genomic-based methods.

## Materials and methods

### Data description

The animal study was approved by the USSES Institutional Animal Care and Use Committee (IACUC). Pedigree records from Polypay flocks participating in the NSIP were merged with historical pedigree records from the USSES beginning in 1970. Pedigree records were traced back until all ancestors were unknown using the Animal Breeders Toolkit (Golden et al., 1992). The depth of pedigree ranged from 1 to 25 generations. The final pedigree consisted of 162,997 individuals born in 193 flocks and included 3,530 sires and 29,489 dams.

### Pedigree analyses

#### Subgroups

**FIGURE 2:**
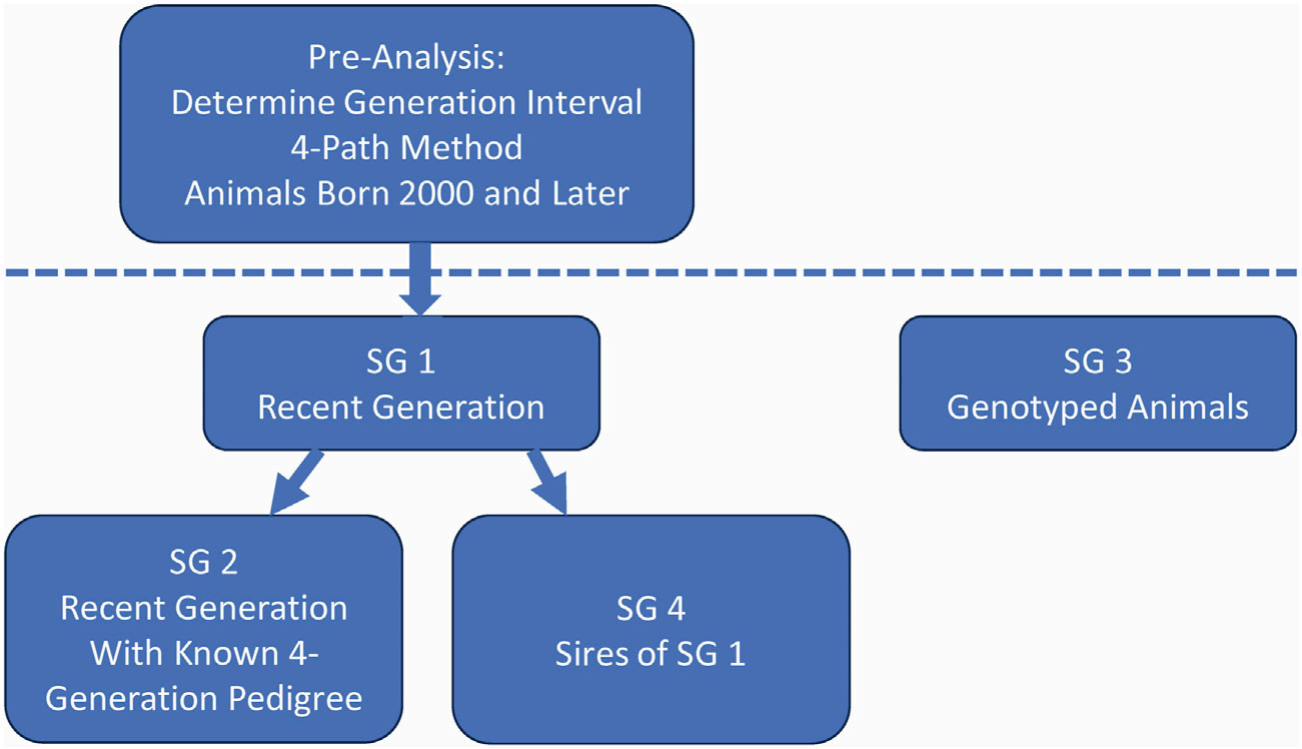
Formation of subgroups used in U.S. Polypay pedigree analyses.

Pedigree analyses were performed using ENDOG software (Gutiérrez and Goyache, 2005). In addition to the complete pedigree, four subgroups were established (Figure 2). Subgroup 1 (SG 1) was defined as the current generation (born 2000–2022). Subgroup 2 (SG 2) was defined as the animals from SG 1 with a minimum of a complete four-generation pedigree. Subgroup 3 (SG 3) included only animals born between 2011 and 2023 that were genotyped with the GGP Ovine 50K BeadChip with a minimum of known parents in the pedigree and which passed the quality control for inclusion in the genomic analyses. Subgroup 4 (SG 4) was defined as the sires of the animals in SG 1. SG3 included 80 sheep that were also in SG 4.

#### Generations

Using the 4-path method for computing generation interval, the mean generation interval was computed as the average of the pathway between sire-son, sire-daughter, dam-son, and dam-daughter (James, 1977; Hill, 1979). Pedigree quality was assessed through the mean maximum number of generations, mean number of complete generations, and the equivalent complete generations for the full population and each subgroup. The mean number of maximum generations was computed as the number of generations between an animal and its furthest ancestor. Mean complete generations was the number of generations between an animal and its furthest generation where all ancestors were known (Gutiérrez and Goyache, 2005). The equivalent complete generations were computed as the sum of (1/2)^n^, where n is the number of generations separating the individual from each known ancestor (Maignel et al., 1996).

#### Inbreeding and relatedness

Relatedness among the animals was measured through the pedigree-based coefficient of inbreeding (F), average relatedness (AR), and the rate of inbreeding per generation. Inbreeding coefficients were computed using ENDOG (Gutiérrez and Goyache, 2005) and the methods described by Meuwissen and Luo (1992). The average relatedness was the probability that a randomly chosen allele from the whole population in the pedigree belonged to a given animal (Gutiérrez and Goyache, 2005). Average relatedness can be used to predict the long-term inbreeding of the population, taking into account both inbreeding and coancestry coefficients (Gutiérrez and Goyache, 2005). Average relatedness is twice the mean coancestry between an animal and all animals in the pedigree, including itself (Cervantes et al., 2008). The change in inbreeding over time, ∆F, was computed as follows:

$$∆F=\frac{F_{i}-\;F_{i-1}}{1-\;F_{i-1}},$$

where F_i_ was the average inbreeding coefficient of the *i*th generation. A linear model was fit in R software (R Core Team, 2021) to test if the regression of ∆F on generation (n = 3.1 years) was significantly different from 0.

#### Founders and ancestors

Exploration of founding alleles within this population included the calculation of the effective number of founders (*f*_*e*_), the effective number of ancestors (*f*_*a*_), the genetic conservation index (GCI), and the marginal contributions of ancestors. The effective number of founders was defined as the number of equally contributing founders that would produce the same genetic diversity as observed in the population. This measure was computed using the average relatedness coefficients of the founders as:

$$f_{e}=1/\sum_{k=1}^{f}q_{k}^{2},$$

where *q*_*k*_ was the probability of gene origin of founder *k*, and *f* was the total number of founders (Lacy, 1989; Gutiérrez and Goyache, 2005). The effective number of ancestors includes the minimum number of ancestors that explain the genetic diversity of the population. The ancestors may but were not required to have been from the founder population and were chosen based on their expected genetic contribution. This value was computed as:

$$f_{a}=1∕\sum_{j=1}^{a}p_{j,}^{2}$$

where *p*_*j*_ was the marginal contribution of ancestor *j*, and *a* was the total number of ancestors (Boichard et al., 1997). The ratio of *f*_*e*_ to *f*_*a*_ determines the presence of genetic bottlenecks, where larger values are associated with more narrow bottlenecks (Boichard et al., 1997). The GCI was developed by Alderson (1992) and quantifies the proportion of genes from each founder in the pedigree of each animal. The GCI was developed to maximize genetic diversity by each founder contributing equally to each animal. Animals with higher GCI, therefore, were expected to have more genetic diversity. A limitation of the use of GCI is the value becomes more informative as the depth of pedigree increases; thus, comparisons across different pedigree depths cannot be made. Accumulated marginal contributions by number of ancestors were plotted. The top 10 marginal contributors were examined further. The number of ancestors contributing to 50% of the genetic diversity of the population was determined.

#### Effective population size

Values representing N_e_ were calculated in ENDOG in seven unique ways. Because N_e_ is based on an idealized population, which does not exist in livestock breeding programs, computing N_e_ using multiple methods that provide a range of estimates is useful. The N_e_ was computed using the individual increase in inbreeding. Additional N_e_ estimates were computed based on the increase in individual inbreeding over maximum generations, complete generations, and equivalent complete generations traced (Gutiérrez et al., 2008; Gutiérrez et al., 2009). Another estimate of N_e_ was obtained from the regression of the inbreeding coefficients on year of birth (Gutiérrez et al., 2003). Because generations were not discrete, N_e_ also was estimated from the log regression of (1–F) on generation number (Pérez-Enciso, 1995). Lastly, N_e_ was estimated from the increase in coancestry for all pairs of individuals (Cervantes et al., 2011). Due to computational limitations, a random subset of 10,000 animals from the full population was selected using R software for computing N_e_ from an increase in coancestry.

#### Population structure

Population structure was determined using flocks to compute Nei’s minimum distance (Nei, 1978) and Wright’s fixation index (F_ST_) (Wright, 1951; Caballero and Toro, 2000; 2002). The Nei’s minimum distance (D) was computed as follows:

$$D_{\text{ij}}=\left[\left(f_{\text{ii}}+f_{\text{jj}}\right)/2\right]‐\;f_{\text{ij}},$$

where *f*_*ii*_ and *f*_*jj*_ were the average coancestry within populations *i* and *j*, and *f*_*ij*_ was the average coancestry between the two populations. Wright’s F_ST_ was computed as follows:

$$F_{ST}=\left(\bar{f}-\;\tilde{f}\right)\;/\;\left(1-\;\tilde{f}\right),$$

where $\bar{f}$ was the average coancestry for the subpopulation, and $\tilde{f}$ was the mean coancestry for the entire population.

### Quantitative analyses

EBVs for SG 4 were obtained from the NSIP using data collected from 1985 to 2022. The EBVs for weaning weight, maternal weaning weight, number of lambs born, and number of lambs weaned, as well as the U.S. Maternal Index encompassing these four traits, were evaluated. The U.S. Maternal Index places positive emphasis on weaning weight, maternal weaning weight, and number of lambs weaned while placing slightly negative emphasis on the number of lambs born, thereby identifying ewes with the genetic capacity to rear all lambs to which they give birth.

### Genomic analyses

#### Quality control

Animals from NSIP-enrolled flocks were genotyped with the GGP Ovine 50K BeadChip (Neogen, Lincoln, NE, United States), which included 51,867 markers. After filtering for animals with unknown parentage (n = 13) and animal call rate < 0.90 (n = 22), 1,856 animals remained for subsequent analyses. These animals came from 32 flocks (1–462 animals per flock). There were 342 males and 1,515 females with birth years ranging from 2011 to 2023. There were 14 flocks with 20 or more genotyped animals; 10 of those flocks had 40 or more. Quality control measures were applied using PLINK software (Purcell et al., 2007); only SNP markers that were mapped to the sheep genome (Oar_v3.1) were retained, and a marker call rate of > 0.90 was applied, leaving 48,360 autosomal SNPs. To account for linkage disequilibrium (LD) among markers, PLINK was used to randomly select a marker every 30 kb based on the estimated rate of decay of LD in the Polypay breed (Zhang et al., 2013). There were 30,995 markers remaining in the “full SNP” dataset. For some analyses, the markers were further filtered for minor allele frequency (MAF) < 0.01, leaving 29,559 markers in the “reduced SNP” dataset. For the N_e_ computation, an additional 50% thinning of the dataset was applied randomly, leaving 14,833 markers in the “thinned SNP” dataset.

#### Genetic diversity metrics

Minor allele frequency categories were defined in increments of 0.05 using PLINK with the full SNP dataset (Brito et al., 2015). Expected heterozygosity (H_E_) and observed heterozygosity (H_O_) were computed using the reduced SNP dataset in PLINK. From these results, the Wright’s inbreeding coefficient, F, was computed as follows:

$$F=\left(H_{E}-H_{0}\right)/H_{E}.$$

The genomic-based N_e_ estimate was computed for the thinned SNP dataset in the NeEstimator software package (Do et al., 2014) using the LD method (Waples and Do, 2008; Do et al., 2014).

The runs of homozygosity (ROH) were determined using the DetectRUNS R package (Biscarini et al., 2019) with the full SNP dataset. The ROH were detected with a sliding window of 50 SNPs with a minimum length of 1,000 kb (equivalent to ∼30 SNPs), a maximum distance allowed between SNPs within an ROH of 250kb, a minimum of 30 SNPs, allowing for one missing SNP, and allowing a maximum of one possible heterozygous SNP (allowing a genotype error rate of 3%) within the defined window with a window threshold of 0.05. The ROH class categories were determined as 1 to 6, > 6 to 12, > 12 to 24, > 24 to 48, and > 48 Mb pairs. The number of ROH per animal was calculated. The inbreeding based on ROH (F_ROH_) was computed as the total length of the genome covered by ROH divided by the total length of the genome covered by SNPs (after quality control). The F_ROH_ was also computed by ROH class category to evaluate past versus new inbreeding. Pearson correlation coefficients were computed between the three measures of inbreeding for the genotyped animals (pedigree-based F, Wright’s inbreeding, and F_ROH_).

Linkage disequilibrium was computed using the r^2^ method described by Hill and Robertson (1968) and implemented in PLINK software using the reduced SNP file. As described by Brito et al. (2015), SNP pairs were assigned to bins based on pairwise marker distance, and the average of each bin was plotted to illustrate the LD decay with increasing physical distance. The distance and LD (r^2^) between adjacent SNP markers were computed.

#### Population structure

Population differentiation was assessed using three methods: the fixation index (F_ST_), principal component analysis (PCA), and model-based population structure. The F_ST_ was computed between 14 flocks with more than 10 genotyped animals using the StAMPP R package (Pembleton et al., 2013) on the reduced SNP dataset. Bootstrapping (n = 100) produced 95% confidence intervals around pairwise F_ST_ values.

The PCA included 14 flocks with more than 10 genotyped animals. To reduce bias for number of animals included per flock, subsets of 20 animals were selected from the 10 flocks with more than 20 genotyped animals; all genotyped animals were included for the four flocks with 10–20 genotyped animals. Subsets of samples were selected with replacement by flock for five replicates using R software, resulting in 262 animals for each PCA. A distance matrix for each replicate was computed using PLINK with the reduced SNP dataset, and the eigenvectors were extracted. Combinations of principal components (PC) 1, 2, and 3 were visualized by flock using R.

The model-based population structure was determined with the reduced SNP dataset for all genotyped animals using ADMIXTURE software (Alexander et al., 2009). ADMIXTURE used the genotype matrix to identify ancestral populations and then assigned animals proportionally to those populations based on allele frequencies. ADMIXTURE was run for 2 to 20 subpopulations (*K*). Alexander et al. (2009) recommended determining the number of *K* using the lowest cross-validation error compared to other *K* values. After 20 *K*, which is beyond the number of ancestral populations expected for a single breed, the cross-validation error was still decreasing. Therefore, the relative decrease in cross-validation error was used to inform the number of *K*. The CLUMPP program was used to align and merge the replicates of the coancestry coefficient matrix, *Q*, produced by ADMIXTURE (Jakobsson and Rosenberg, 2007). Then, STRUCTURE PLOT was used to generate bar plots to visualize each animal by flock (Ramasamy et al., 2014).

## Results

### Pedigree analyses

The generation interval was 3.13 ± 0.02 years based on the weighted mean of each path: sire-son (2.97 ± 0.10 years), sire-daughter (3.12 ± 0.12 years), dam-son (3.45 ± 0.05 years), and dam-daughter (3.12 ± 0.04 years). Based on these results, the SG 1 and SG 2 populations included animals born in the most recent generation, from 2020 to 2022. Summary statistics for the full population and subgroups are presented in Table 1.

**TABLE 1:** Summary statistics for the full population and subgroups 1, 2, 3, and 4 for U.S. Polypay sheep.

Parameter	Full population	SG 1^a^	SG 2^b^	SG 3^c^	SG 4^d^
N	162,997	20,701	12,685	1,856	509
Flocks	193	77	57	32	69
F^e^, %	2.19	3.48	5.29	2.94	4.42
AR^f^, %	1.11	1.96	2.45	1.78	2.07
Mean maximum generations (maximum)	9.6 (25.0)	18.1 (24.0)	19.8 (24.0)	17.3 (24.0)	15.2 (23.0)
Mean complete generations (maximum)	2.3 (7.0)	3.2 (7.0)	4.4 (7.0)	3.7 (7.0)	3.4 (7.0)
Equivalent complete generations (maximum)	4.6 (12.5)	7.6 (12.2)	9.3 (12.2)	7.8 (11.9)	7.2 (11.5)
*f*_*e*_ ^g^	271	153	100	163	131
*f*_*a*_ ^h^	144	58	37	64	48
*f*_*e*_ */f*_*a*_	1.9	2.6	2.7	2.5	2.7
GCI^i^	10.9	16.4	23.6	22.9	16.9
Number of ancestors explaining 50% of genetic variation	79	22	14	24	19

^a^ SG 1, Subgroup 1, which included animals born from 2020 to 2022.

^b^ SG 2, Subgroup 2, which included SG 1 with a minimum 4-generation pedigree.

^c^ SG 3, Subgroup 3, which included genotyped animals.

^d^ SG 4, Subgroup 4, which included sires of SG 1.

^e^ F, average individual coefficient of inbreeding.

^f^ AR, average relatedness.

^g^ *f*_*e*_, effective number of founders.

^h^ *f*_*a*_, effective number of ancestors.

^i^ GCI, genetic conservation index.

**FIGURE 3:**
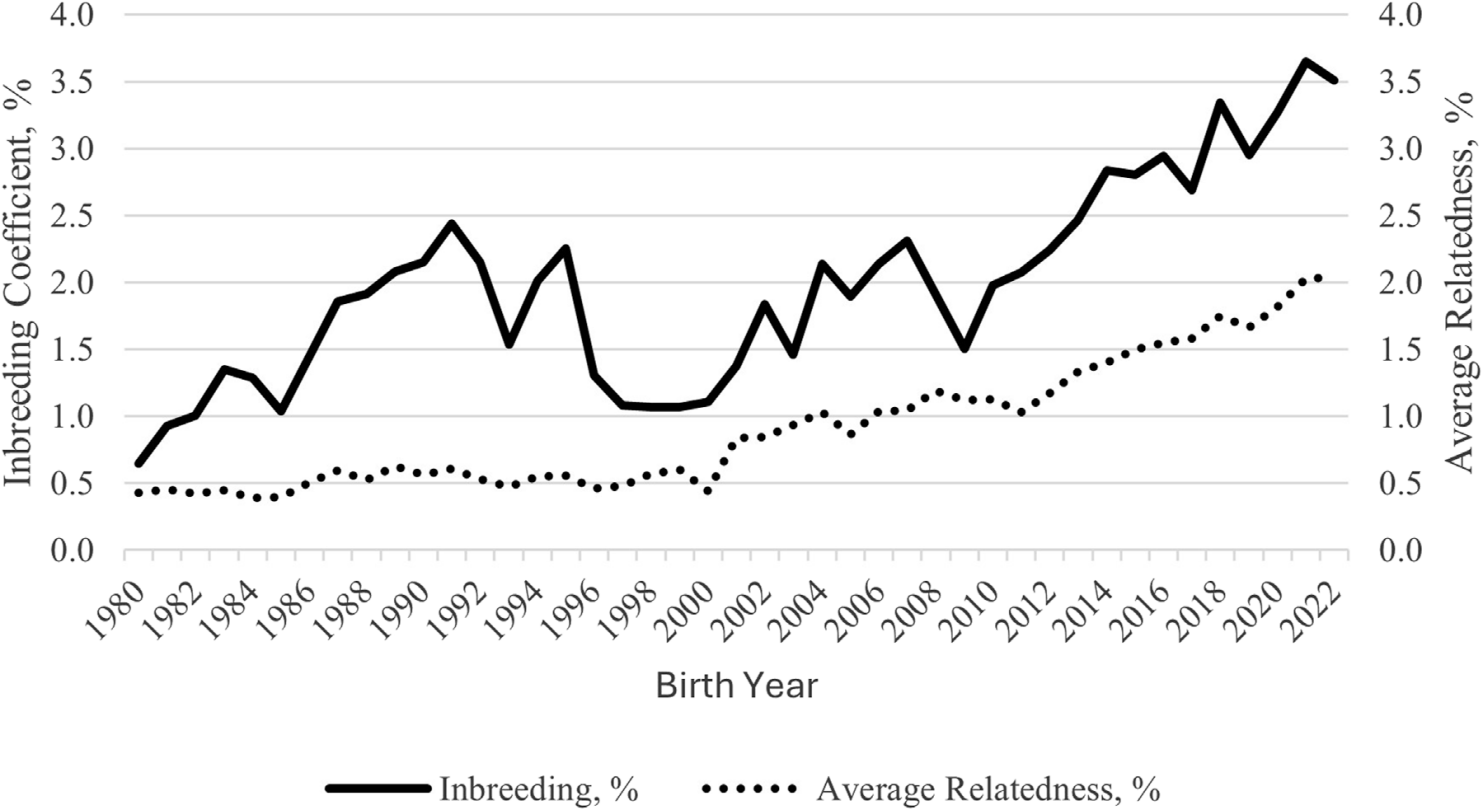
Inbreeding and average relatedness trends from 1980 to 2022 for U.S. Polypay sheep.

Overall F and AR were low for the Polypay breed. Among these analyses, their values were highest for SG 2, which included the most complete pedigrees. Trends in F and AR since 1980 were plotted (Figure 3). For the generation interval of 3.1 years, the ∆F was 0.215% per generation and did not differ across generations (*p* = 0.95). The *f*_*e*_, *f*_*a*_, and *f*_*e*_/*f*_*a*_ for the full population were 271, 144, and 1.9, respectively (Table 1).

**FIGURE 4:**
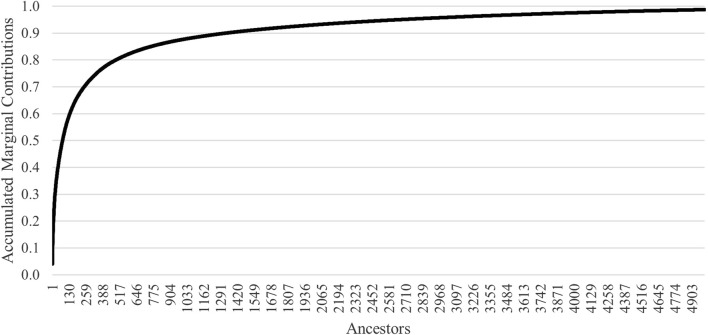
Accumulated marginal contributions by the number of ancestors for U.S. Polypay sheep.

Accumulated marginal contributions of ancestors demonstrate that 50% of the genetic variation in the population was attributed to 79 ancestors (Figure 4). The top 10 marginal contributors were all rams originating from eight flocks that contributed 20.3% of the genetic variation in the population (Table 2). Three of the eight flocks participated in genotyping of their animals. Only one ram was from the founding flock, and the most recent influential ram was born in 2006.

**TABLE 2:** Top 10 marginal contributors to the U.S. Polypay gene pool.

Animal rank	Marginal contribution	Accumulated contribution	Progeny	Flock	Birth year
1	0.041	0.041	464	A	1991
2	0.031	0.072	379	B	2002
3	0.031	0.103	298	C	1993
4	0.017	0.120	377	D	1975
5	0.016	0.136	153	A	1992
6	0.015	0.150	28	E	1992
7	0.014	0.164	229	A	1994
8	0.013	0.178	184	F	2004
9	0.013	0.191	182	G	1999
10	0.012	0.203	323	H	2006

The pedigree-based estimates of N_e_ ranged from 41 (based on the increase in F by complete generation) to 249 (based on the increase in F by maximum generation) (Table 3). Population differentiation between the flocks was low for both Nei’s distance (0.027) and Wright’s F_ST_ (0.027).

**TABLE 3:** Pedigree-based estimates of N_e_ in the U.S. Polypay population.

Method	N_e_ estimate
Increase in F by maximum generation	249
Increase in F by complete generation	41
Increase in F by equivalent generation	81
Individual increase in F	110
Regression on birth year	72
Log regression of (1–F) on generation number	70
Individual increase in coancestry	101

### Quantitative analyses

The range of the NSIP U.S. Maternal Index scores and EBVs for its component traits were examined for the sires in SG 4 (Table 4). After removing animals with accuracies below the minimum reporting thresholds for the NSIP, the average accuracy was 76, 62, 59, and 55 for weaning weight, maternal weaning weight, number of lambs born, and number of lambs weaned EBVs, respectively. Particularly in younger sires, the prediction accuracies were near the lower bound of the reporting threshold.

**TABLE 4:** 2022 NSIP values for the U.S. Maternal Index score, weaning weight (WWT) EBV, maternal weaning weight (MWWT) EBV, number of lambs born (NLB) EBV, and number of lambs weaned (NLW) EBV for U.S. Polypay subgroup 4.

Trait	n	Mean EBV (range)	Mean accuracy (range)
U.S. Maternal Index Score	435	110.1 (86.5–124.0)	
WWT EBV, kg	477	1.2 (−3.0–3.5)	76 (36–96)
MWWT EBV, kg	436	1.3 (−1.7–4.2)	62 (35–95)
NLB EBV, lambs per ewe lambing	444	0.1 (−0.4–0.5)	59 (25–94)
NLW EBV, lambs per ewe lambing	445	0.2 (−0.2–0.4)	55 (20–92)

### Genomic analyses

**FIGURE 5:**
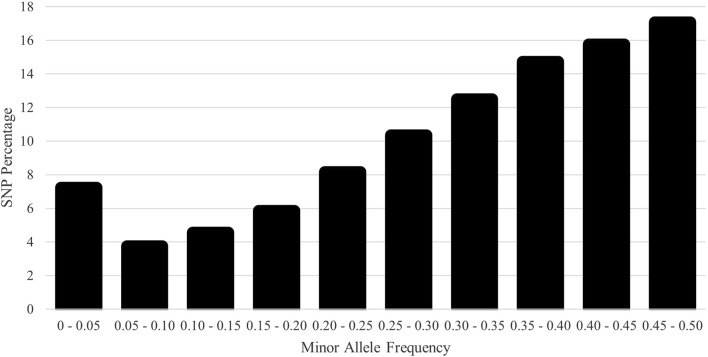
Distribution of single-nucleotide polymorphism (SNP) frequency by the minor allele frequency (MAF) category for the U.S. Polypay sheep.

**FIGURE 6:**
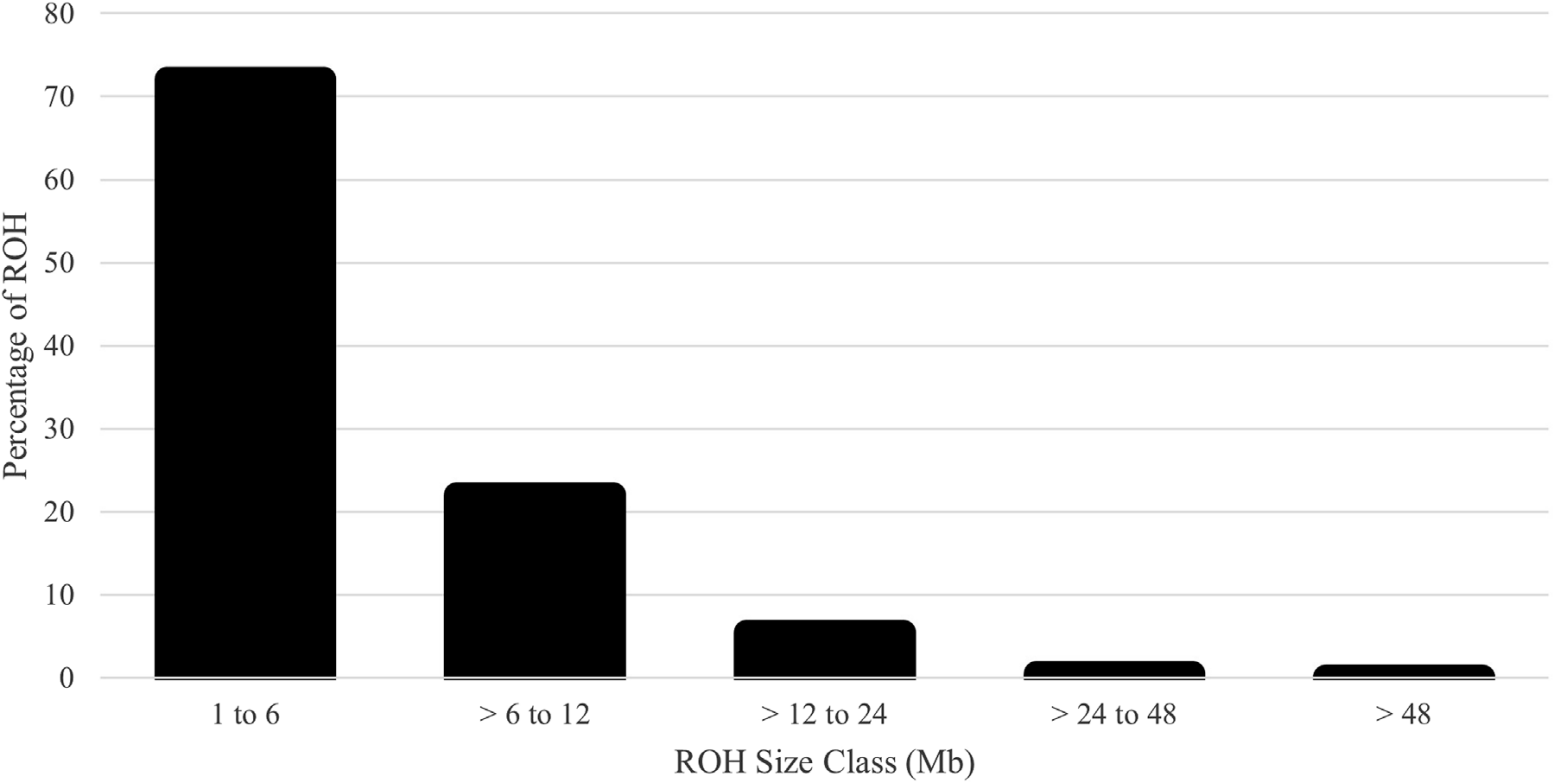
Percentage of runs of homozygosity (ROH) assigned to each size class for U.S. Polypay sheep.

The distribution of MAF was classified in Figure 5 with few fixed and rare SNPs. Most SNPs were highly polymorphic, defined as MAF > 0.30 (60.1%). The H_E_, H_O_, and Wright’s inbreeding were 0.409, 0.403, and 1.3%, respectively. The genomic N_e_ estimate for the current population was 118. The computed mean F_ROH_ was 5.1% (range 0.0%–50.2%). The number of ROH averaged 14.5 per animal with a range of 0–62. The majority of ROH were short; the percentages of ROH by size class are shown in Figure 6.

**FIGURE 7:**
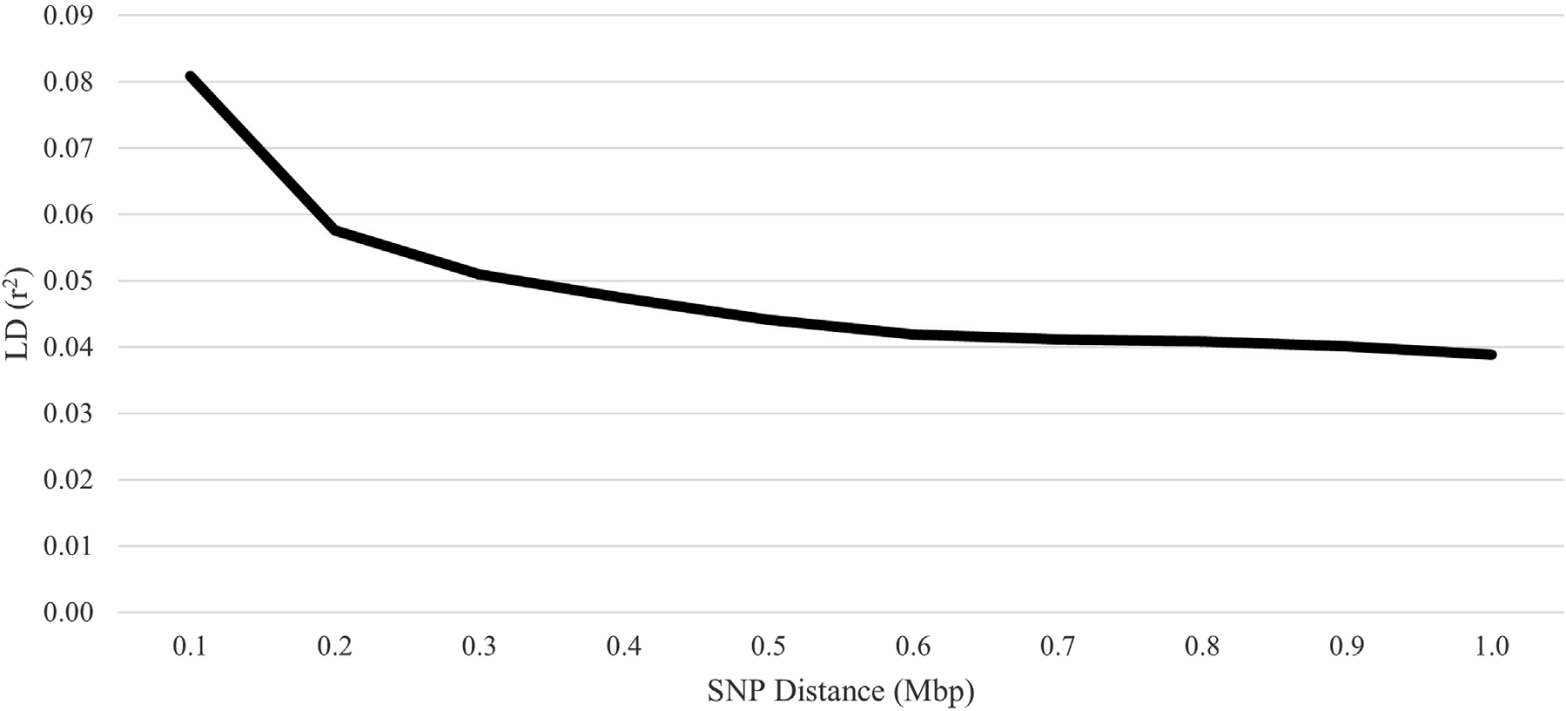
Linkage disequilibrium (LD) decay over increasing single-nucleotide polymorphism (SNP) distance for U.S. Polypay sheep.

The average inbreeding for the genotyped animals was 2.9%, 1.3%, and 5.1% for pedigree-based F, Wright’s F, and F_ROH_, respectively. Inbreeding within each ROH class was 2.9%, 1.5%, 0.5%, 0.1%, and 0.0% for 1 to 6, > 6 to 12, > 12 to 24, > 24 to 48, and > 48 Mb, respectively. The Pearson correlation coefficients between the pedigree-based F and genomic-based F were 0.57 for Wright’s F and 0.52 for F_ROH_. However, the Pearson correlation between the two genomic-based Fs demonstrated congruence (0.87). The overall LD among SNP pairs was low and declined with increasing distance (Figure 7). The average distance between adjacent SNPs was 0.09 Mb, with an average LD of 0.07.

**FIGURE 8:**
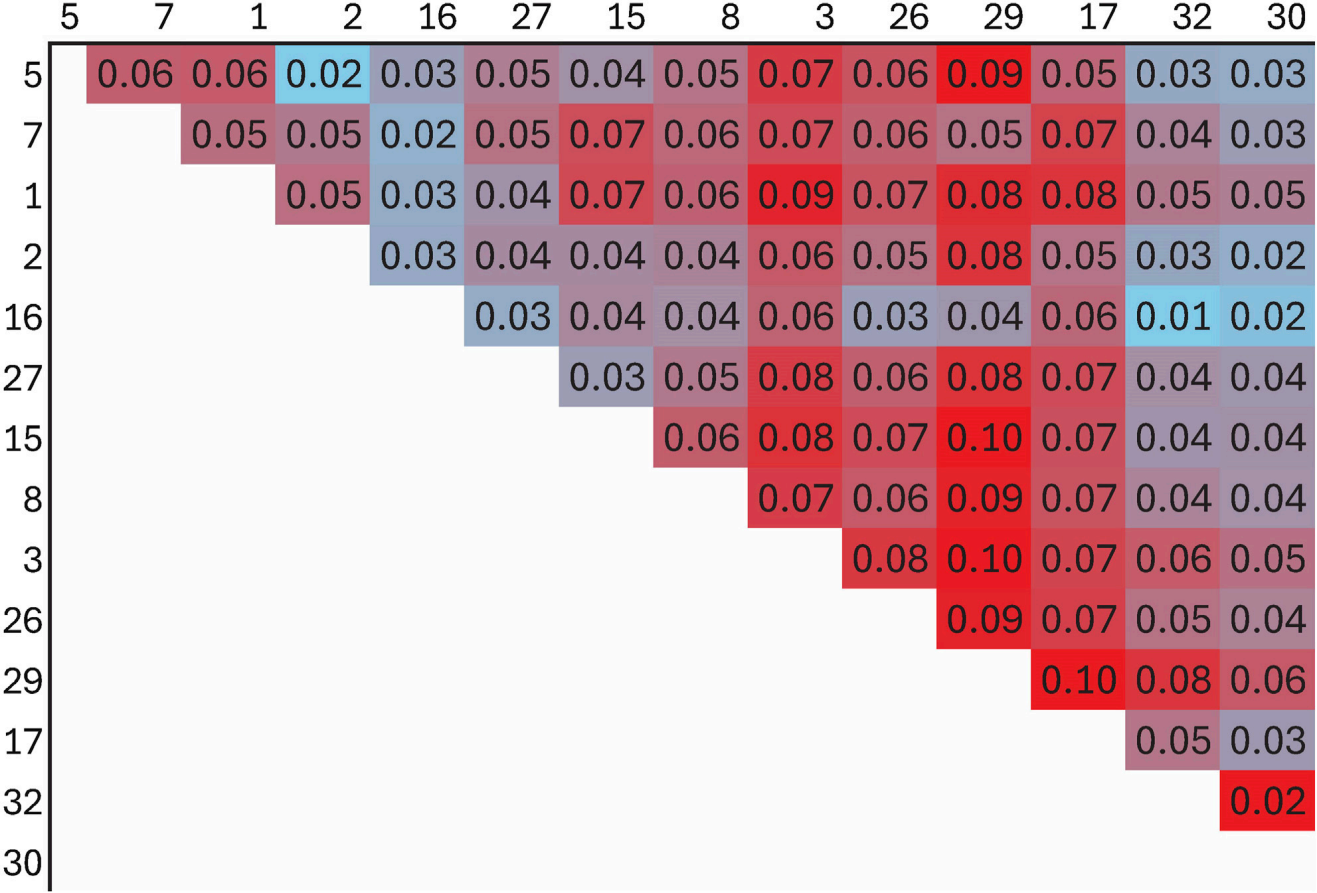
Heat map of fixation index (F_ST_) for flocks with more than 10 genotyped Polypay sheep.

**FIGURE 9:**
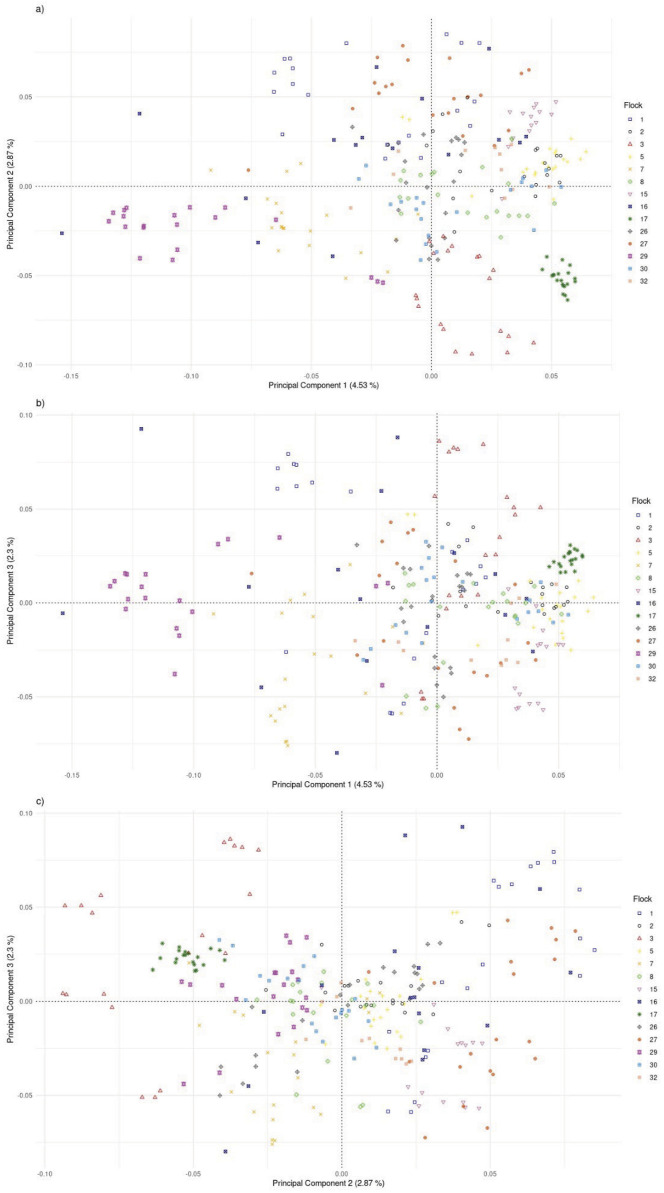
Plot of principal components 1 and 2 **(A)**, 1 and 3 **(B)**, and 2 and 3 **(C)** for U.S. Polypay sheep genotyped from 14 flocks (replicate 1).

**FIGURE 10:**
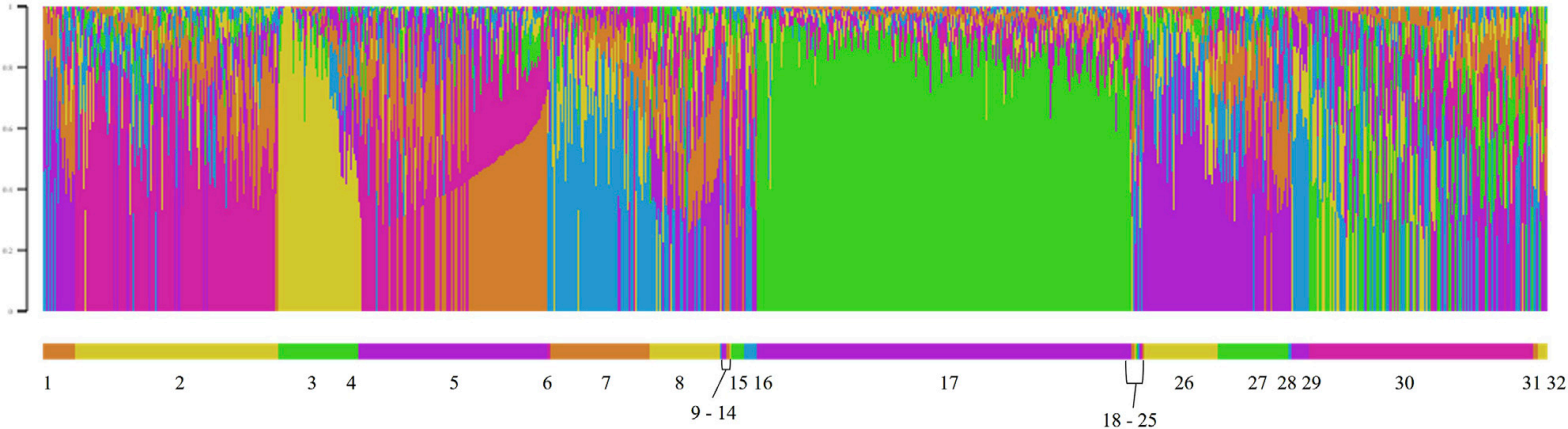
Model-based population structure for *K = 6* for genotyped Polypay sheep, sorted by flock, where Flock 17 is the U.S. Sheep Experiment Station.

The F_ST_ among the 14 flocks ranged from 0.01 to 0.10 with a mean of 0.05 (Figure 8; Supplementary Figure S1). Flock 16 had the lowest average differentiation from other flocks (0.03), while Flock 29 had the most (0.08). The foundation flock, USSES (Flock 17), had a mean F_ST_ of 0.06 and ranged from 0.03 to 0.10 when compared to the other flocks. The three flocks with the highest mean F_ST_ values, Flocks 3, 17, and 29, are differentiated in the PC1 vs. PC2 (Figure 9A; Supplementary Figures S2A, S3A, S4A, S5A) and PC1 vs. PC3 (Figure 9B; Supplementary Figures S2B, S3B, S4B, S5B) plots. The first of five replicates is shown in Figure 9, and replicates 2 to 5 are provided as Supplementary Material. The patterns of the PC visualizations were consistent across all replicates. The variation explained by each PC across replicates was low and ranged from 4.4% to 4.7% for PC1, 2.9%–3.2% for PC2, and 2.2%–2.5% for PC3. The flock with the lowest mean F_ST_ values, Flock 16, was widely dispersed across the PC plots and had extreme outliers in all plots. A model-based population structure was visualized in Figure 10 for all genotyped animals. As with the F_ST_ and PCA, Flocks 3 and 17 show differentiation in the subpopulation assignments. The remaining flocks have an admixed population structure.

## Discussion

The Polypay breed plays an important role in the U.S. sheep industry as a prolific Western U.S. range ewe and throughout the Central and Eastern U.S. in more intensively managed production systems. Continued genetic improvement in this breed will benefit from implementing genomic selection. As such, a thorough understanding of the genetic diversity and population structure was needed to develop sustainable breeding strategies.

### Genetic diversity

The generation interval of 3.1 years was consistent with that of the Canadian Polypay, which is 3.4 years (Stachowicz et al., 2018). Recently reported generation intervals for other U.S. sheep breeds include 2.9 years, 3.1 years, and 2.9 years for Suffolk (Wilson et al., 2022), Targhee (Wilson et al., 2024), and Katahdin (Nilson et al., 2023), respectively. Other reported generation intervals include 3.6 years for the Brazilian Morada Nova (McManus et al., 2019), 3.3 years for the Canadian Suffolk (Stachowicz et al., 2018), and 3.2 years for the German White-headed Mutton sheep (Addo et al., 2021). Pedigree-based inbreeding estimates obtained in the current study in U.S. Polypay (3.5% for SG 1 and 5.3% for SG 2) were similar to that in the Canadian population (3.5%). The rate of inbreeding was higher for the Canadian population than for the U.S. population. The *f*_*e*_ and *f*_*a*_ were higher for the U.S. population, as was the number of ancestors explaining 50% of the gene pool. In comparison with other breeds, U.S. Suffolk and Czech Republic Romanov inbreeding was higher, while German White-headed Mutton sheep and Canadian Suffolk had lower inbreeding (Stachowicz et al., 2018; Vostry et al., 2018; Addo et al., 2021; Wilson et al., 2022).

The genomic inbreeding measures account for Mendelian sampling rather than probabilities with pedigree-based inbreeding and are, therefore, more accurate. However, not all animals can be genotyped, so both measures should be used in combination. Within ROH classes, the most inbreeding was found within the two smallest classes (2.9% for 1–6 Mb and 1.5% for > 6–12 Mb), indicating past rather than recent inbreeding. Even without exact agreement between the three measures at an individual level, inbreeding was low, with no cause for immediate concern. In accordance with the low levels of inbreeding, the AR values were low for the full population and the subgroups. The AR values predict the long-term inbreeding in the population and provide further evidence of the genetic diversity present in the current Polypay breed. The AR was lower than F for the Polypay, which was also observed for Katahdin (Nilson et al., 2023) and Morada Nova (McManus et al., 2019), but not for the Targhee (Wilson et al., 2024). As observed by Cervantes et al. (2008), because the mean F was higher than half of the mean AR, matings occurred between related animals. Fluctuations in F and AR, such as those seen from 1996 to 2000 and in 2009, are expected as new breeders join the NSIP, whose flocks may be less genetically connected to other flocks, or as breeders selectively report data on animals. Based on ∆F, inbreeding has been accumulating but not at an increasing rate. Low F and AR for SG 3 suggest the animals sampled for genotyping were less related than the current generation (SG 1 and SG 2), signifying effective sampling to estimate diversity.

Measures of founding alleles provide an understanding of the flow of genes from the founder population to the current gene pool and quantify any genetic bottlenecks that happened over time. For the full population, the *f*_*e*_/*f*_*a*_ was 1.9, indicating the presence of bottleneck events. The ratio was higher for the subgroups (2.5–2.7), which represent the recent generation(s) of animals. However, only 79 ancestors explain 50% of the current gene pool. For other U.S. sheep breeds, 82 and 46 ancestors explained 50% of the gene pool for Katahdin (Nilson et al., 2023) and Suffolk (Wilson et al., 2022), respectively. The top 10 marginal contributors to the current gene pool were all rams born in 2006 or earlier. They had a range of offspring from 28 to 377, which is consistent for U.S. sheep breeds that typically do not utilize advanced reproductive technologies, such as artificial insemination, on a large scale.

Based on the low F and AR from the rams contributing to the current generation (SG 4) and the lack of recent highly influential sires, there was no evidence of dominant sires or sire lines in the current breeding population. Although the EBVs and Maternal Index for current sires showed a wide range of values, most rams were grouped close to the mean. Increasing the accuracy of breeding value predictions for existing traits through genomic selection, particularly for lowly heritable traits, those observed later in life, or those only expressed in one sex, and increasing the number of economically important traits evaluated in the Polypay breed are essential for breeders to differentiate among rams to meet their specific breeding objectives.

There is no single measure that defines an acceptable level of genetic diversity, but rather, a collective body of evidence describes the genetic variability of the population. As an attempt to provide guidance, the Food and Agriculture Organization of the United Nations (FAO) has recommended a goal of less than a 1% rate of inbreeding per generation and a N_e_ of more than 50 (FAO, 1998). Meuwissen (2009) provided a more conservative recommendation of a N_e_ of 100. Leroy et al. (2013) computed N_e_ for 140 breeds from four species using multiple methods. They concluded the species and population structure should be considered when determining which method to use and the minimum acceptable N_e_. There is general agreement that pedigree-based methods for N_e_ estimation are to be considered in context as a range of estimates rather than a precise value. The rate of inbreeding was 0.215 per generation, which is well below the level of concern. Similarly, six of the seven pedigree-based N_e_ estimates were above 50, and three were above 100. The genomic-based N_e_ estimate of 118 exceeded the threshold level.

The majority of the SNPs were highly polymorphic, and both H_E_ and H_O_ levels were consistent with other studies using the GGP Ovine 50K, including the Czech Sumava (H_E_: 0.43; H_O_: 0.42), Czech Wallachian (H_E_: 0.40; H_O_: 0.41) (Machová et al., 2023), and Slovenian Valachian (H_E_: 0.39–0.43; H_O_: 0.39–0.43) (Mészárosová et al., 2022), and were higher than the U.S. Katahdin (H_O_: 0.37) (Becker et al., 2022). Because the Polypay is a composite breed with a relatively short history compared to other sheep breeds, high levels of heterozygosity were expected. In addition to being consistent with other sheep breeds, the majority of ROH were short, indicative of past inbreeding and founder effects rather than more recent inbreeding. The majority of ROH (72.1%) were in the smallest ROH class (1–6 Mb) compared to 95.9% in the smallest class (1–5 Mb) for U.S. Suffolk (Wilson, 2023), 88.2% in the smallest class (1–6 Mb) for 14 South African sheep breeds (Dzomba et al., 2021), the majority in the two smallest classes (<10 Mb) for six Irish commercial breeds (Purfield et al., 2017), and 71%–86% in the two smallest classes (<4 Mb) for 17 global sheep populations from eight breeds (Selli et al., 2021).

Successful implementation of genomic selection depends on both pedigree linkages among animals with both phenotypic and genomic data and breeding candidates and LD between the markers and the quantitative trait loci (QTL) of traits of interest (Meuwissen et al., 2001; Daetwyler et al., 2012; Wientjes et al., 2013). Based on the markers studied, the average LD between markers in the Polypay was low at all distances, and the LD declined with increased distance between markers. This is consistent with the findings of Zhang et al. (2013), who reported the lowest LD for Polypay of the five breeds evaluated. The LD decay closely mirrored New Zealand Romney, Coopworth, and Perendale (Prieur et al., 2017) and Qezel and Australian Suffolk, but LD was higher for Soay, Border Leicester, and Barbados Blackbelly (Kijas et al., 2014). Genome-wide association studies using a 50K BeadChip will suffer from low power due to poor genome coverage coupled with small blocks of LD in sheep. Even so, substantial improvements in the accuracy of estimated breeding values are anticipated using a moderately dense array due to a more reliable genomic relationship matrix.

### Population structure

Genomic assessment of the 14 flocks with the most animals genotyped allowed a finer scale comparison of flocks. Some flocks (2, 16, 30) showed high levels of admixture through low F_ST_ values (<0.05), dispersion throughout the PC, and assignment to multiple ancestral populations based on the model-based population structure. Although Flock 16 had the lowest level of F_ST_ differentiation and high dispersion across the PC plots, there were several outliers in the PC plots, suggesting selection for an extreme phenotype or, more likely, the introduction of another breed. Flocks 3, 17, and 29 had distinct population structures with higher F_ST_, separation in the PC, and distinctly different subpopulation assignments in the model-based population structure.

Differentiation among flocks provides a safeguard to maintain genetic diversity within the Polypay breed. However, ensuring sufficient genetic connectedness exists among flocks is important for genetic evaluation (Lewis et al., 2005; Kuehn et al., 2007; Kuehn et al., 2008; Kuehn et al., 2009). Adequate sampling of animals and flocks for the formation of optimal reference populations for genomic selection also needs to be prioritized.

The USSES flock (Flock 17), in which the Polypay breed was developed, had the most uniform clustering in the PC and the highest proportional assignment to a single subpopulation in the model-based population structure. The only flock with F_ST_ less than 0.05 is another national research flock, the U.S. Meat Animal Research Center (USMARC; Flock 30), which received a portion of its ewes and rams from USSES. The national flocks are intentionally sharing genetics to develop genetic reference flocks (GRF) that will serve sheep producers by incorporating industry sires into the research flocks. Continued exchange of genetics among national flocks will create opportunities to study genetics by environment by management interactions. Other than USMARC, substantial differentiation has occurred as the remaining flocks have moved toward a more intensive production and management environment. Meanwhile, the USSES has favored a moderately prolific range ewe with an emphasis on twins in a once-per-year lambing system.

In composite breed development, it is presumed that the initial percentages of the breeds combined will persist over time. This can be evaluated by comparing the genomes of founder breeds of the composite breed to identify the ancestral origins of regions throughout each chromosome using software such as ChromoPainter (Lawson et al., 2012; Paim T.d. P. et al., 2020; Paim T. D. P. et al., 2020). Using these methods, Paim T. D. P. et al. (2020) evaluated the breed composition of Brangus cattle, which was expected to be 62.5% Angus and 37.5% Brahman. They found some chromosome regions with directional selection for the Angus breed and fewer for the Brahman breed. The current Brangus population was determined to be 70.4% Angus. Further analysis of Polypay sheep using these methods is warranted to evaluate how selection and use in different production systems have influenced the retained breed percentages over time. Selection for prolificacy in an accelerated lambing system may favor a higher percentage of chromosomal regions from the high prolificacy of Finnsheep and the aseasonality of Dorset. A comparison of animals from the admixed flocks and the foundation flock would be of particular interest.

## Conclusion

There is substantial genetic diversity in the Polypay breed and population structure. These findings show that the breed has become more diverse from its foundation flock, likely due to its popularity and incorporation into the sheep industry. As the industry moves toward the implementation of genomic selection, low levels of inbreeding, high levels of heterozygosity, and large N_e_ are beneficial for the Polypay breed. Population structure and differentiation among flocks provide an additional safety net for maintaining the genetic diversity in the breed. These baseline levels of genetic diversity and population structure should be re-evaluated in 10–15 years after genomic selection is implemented. Because breeders want to increase the proportion of favorable alleles in a population and subsequently decrease unfavorable alleles, we expect to observe changes in the genetic makeup of the population over time. Selection is expected to decrease genetic diversity in the breed, but the gains in genetic improvement have the opportunity to bring about positive changes in this economically important breed.

## Data Availability

The data analyzed in this study are subject to the following licenses/restrictions: The raw data cannot be made available as they are the property of the sheep producers participating in the NSIP, and this information is commercially sensitive. Requests to access these datasets should be directed to carrie.wilson@usda.gov.

## Supplementary material

SUPPLEMENTARY FIGURE S1 Heat map of fixation index (F_ST_) (upper diagonal) and 95% confidence intervals (lower diagonal) for flocks with more than 10 genotyped Polypay sheep.

SUPPLEMENTARY FIGURE S2 Plot of principal components 1 and 2 **(A)**, 1 and 3 **(B),** and 2 and 3 **(C)** for Polypay sheep genotyped from 14 flocks (replicate 2).

SUPPLEMENTARY FIGURE S3 Plot of principal components 1 and 2 **(A)**, 1 and 3 **(B)**, and 2 and 3 **(C)** for Polypay sheep genotyped from 14 flocks (replicate 3).

SUPPLEMENTARY FIGURE S4 Plot of principal components 1 and 2 **(A)**, 1 and 3 **(B)**, and 2 and 3 **(C)** for Polypay sheep genotyped from 14 flocks (replicate 4).

SUPPLEMENTARY FIGURE S5 Plot of principal components 1 and 2 **(A)**, 1 and 3 **(B)**, and 2 and 3 **(C)** for Polypay sheep genotyped from 14 flocks (replicate 5).
