# Supplementary figures and images for: Assessment of genetic diversity and population structure of U.S. Polypay sheep from breed origins to future genomic selection
Source: Front Genet. 2024 Aug 5;15:1436990. doi: 10.3389/fgene.2024.1436990 (PMC11330798; doi:10.3389/fgene.2024.1436990)

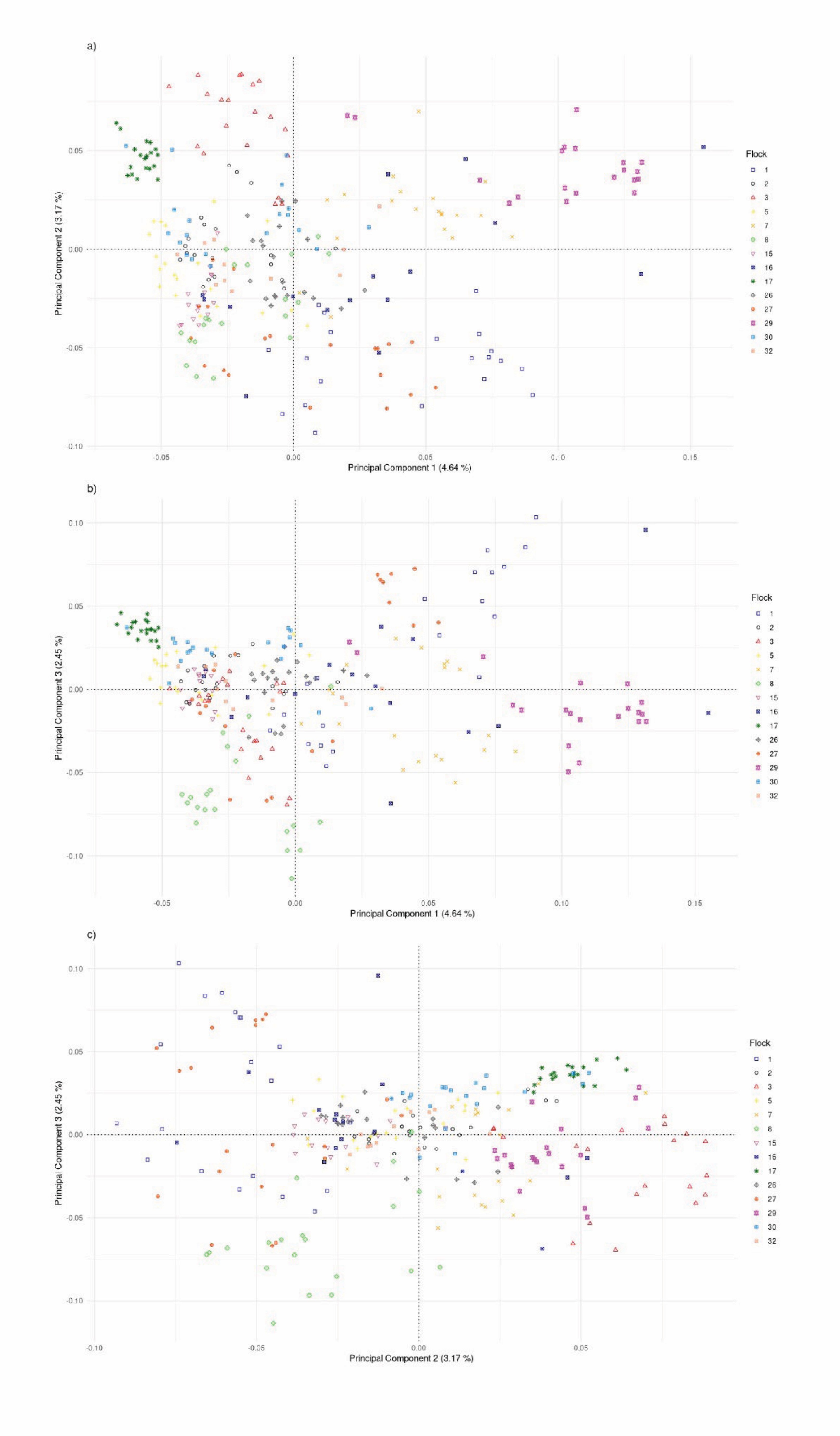

Supplement: Supplementary file 1 [file Image3.JPEG]

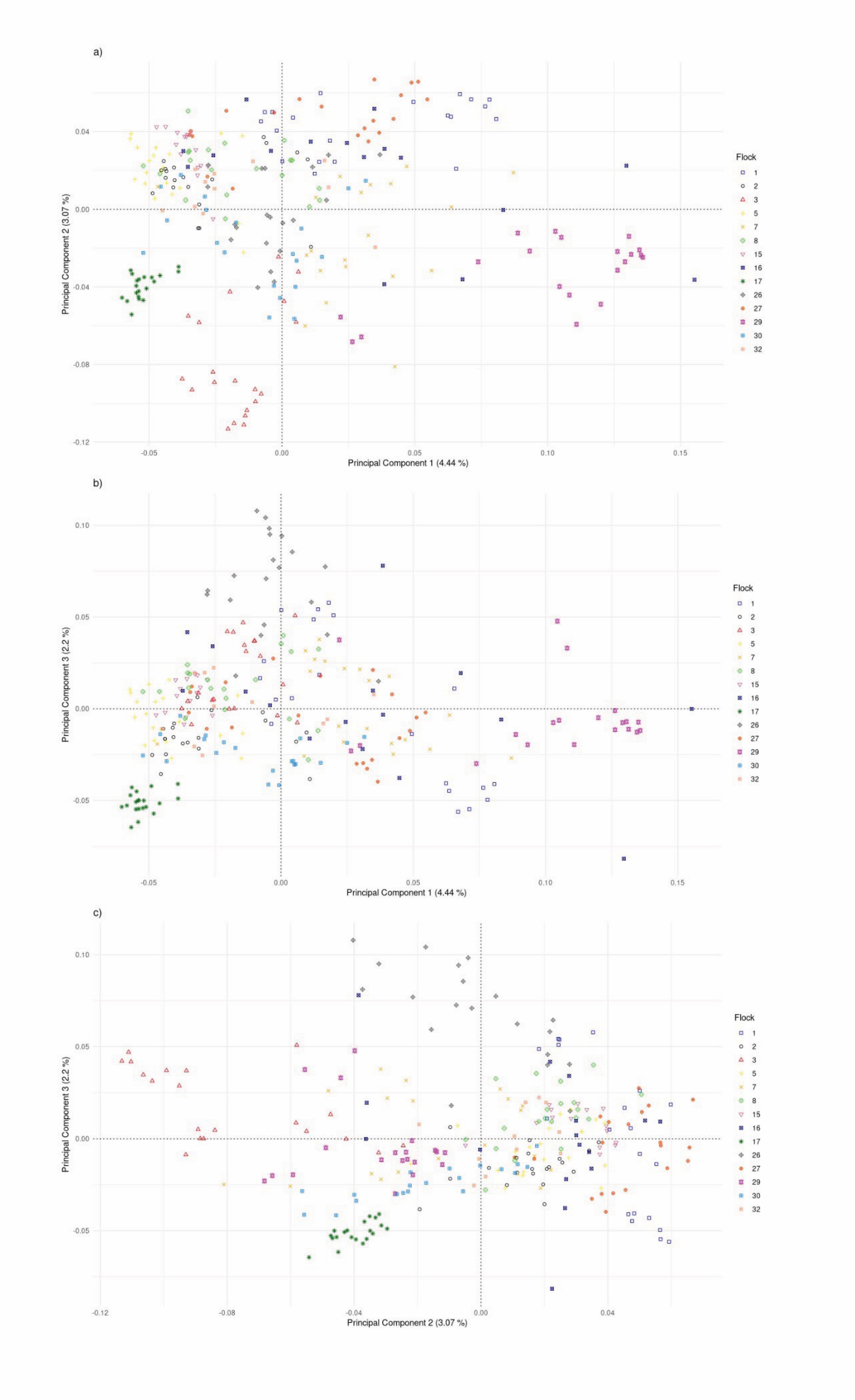

Supplement: Supplementary file 2 [file Image1.JPEG]

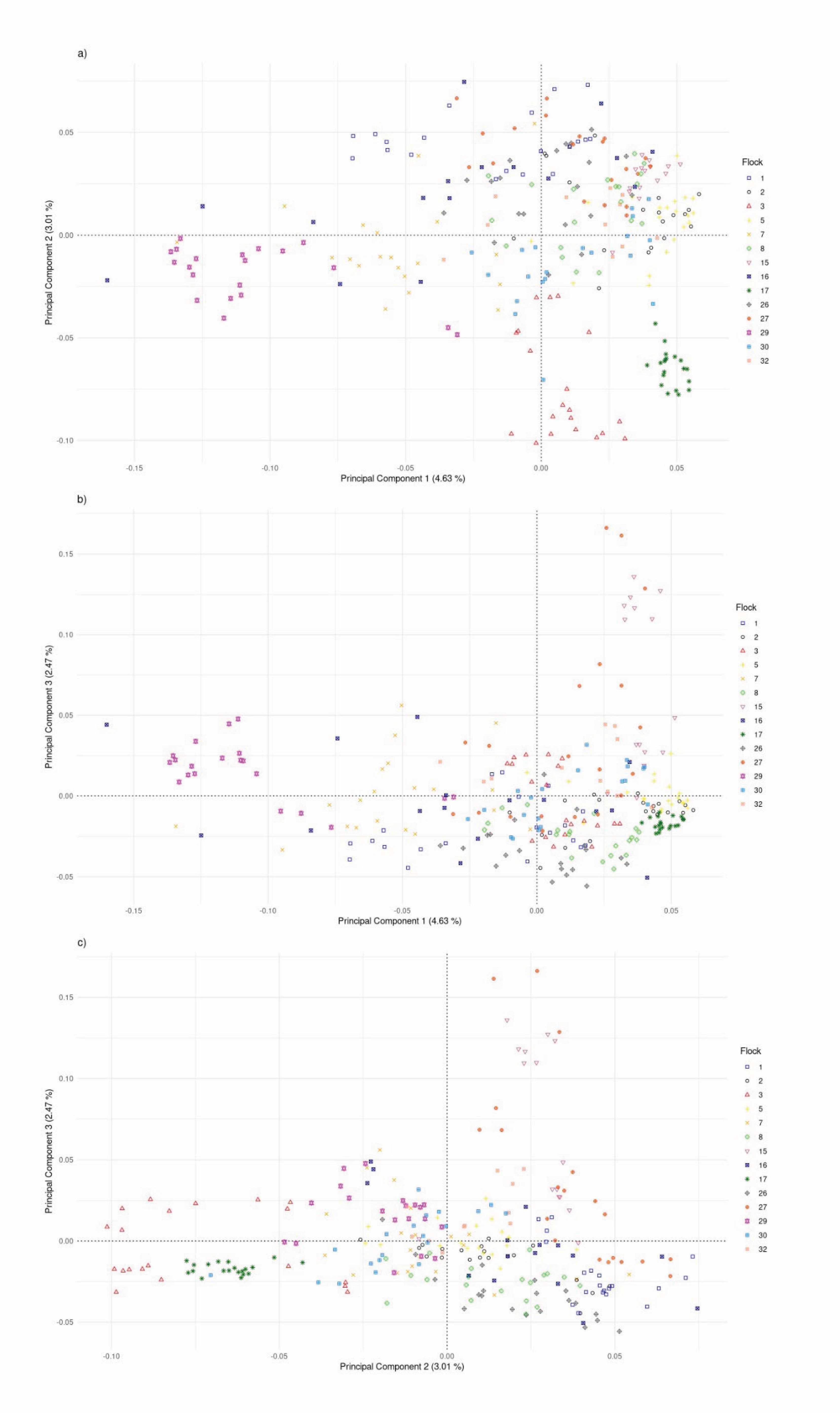

Supplement: Supplementary file 3 [file Image4.JPEG]

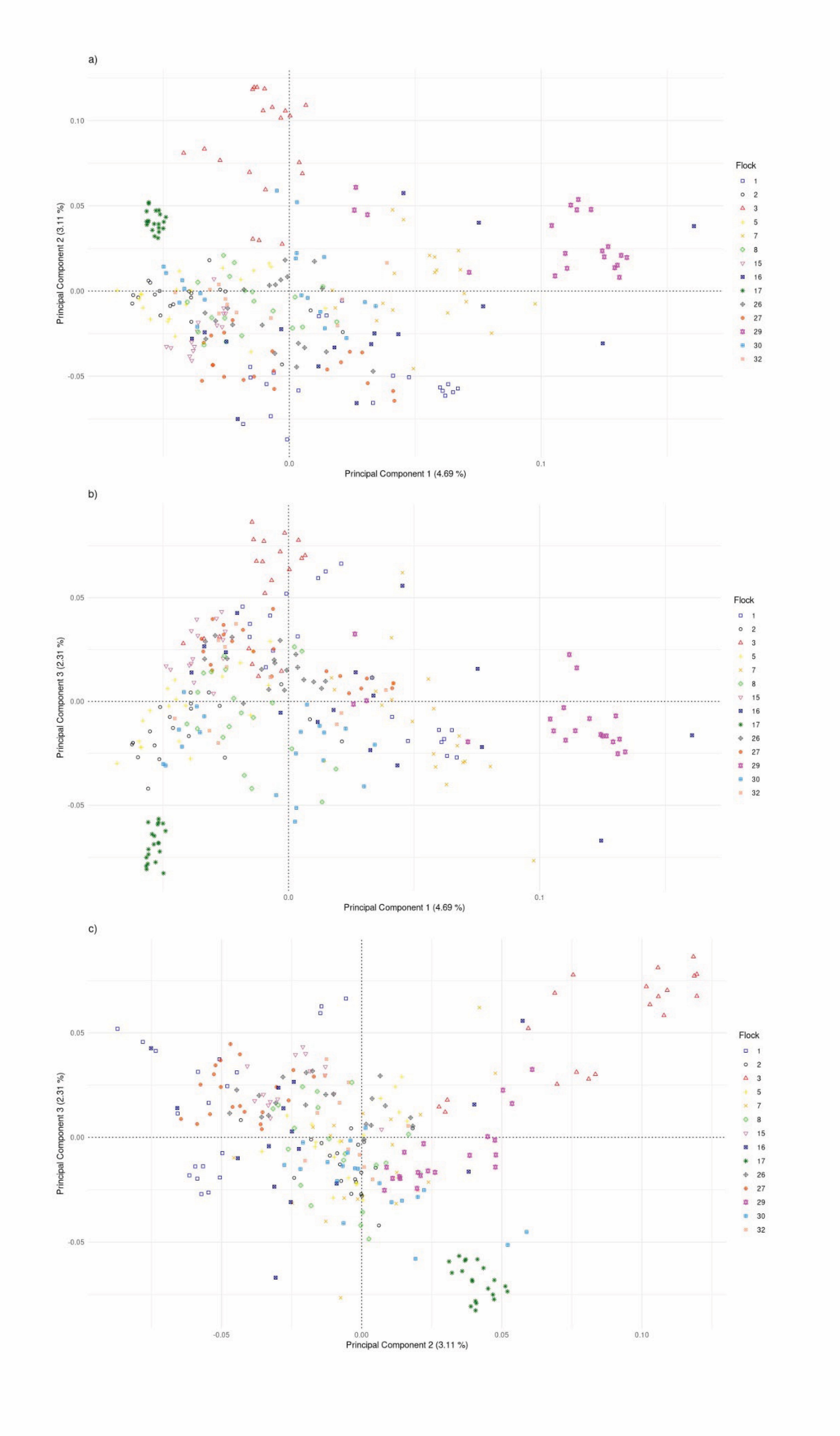

Supplement: Supplementary file 4 [file Image2.JPEG]

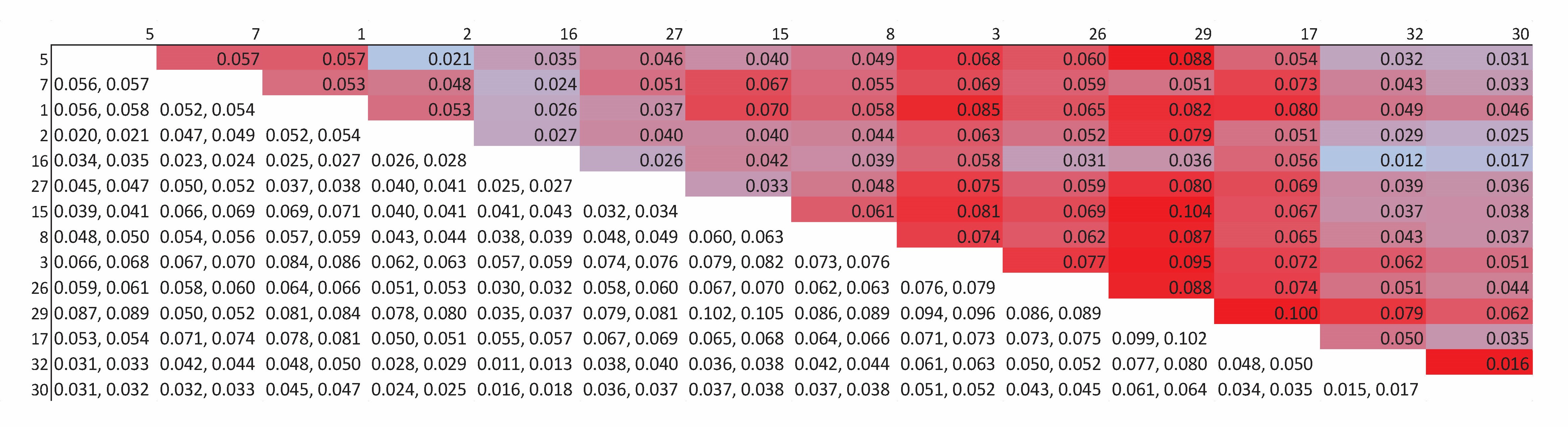

Supplement: Supplementary file 5 [file Image5.JPEG]
